# Supplementary material for: Memes: A motif analysis environment in R using tools from the MEME Suite
Source: PLoS Comput Biol. 2021 Sep 27;17(9):e1008991. doi: 10.1371/journal.pcbi.1008991 (PMC8496816; doi:10.1371/journal.pcbi.1008991)
Supplement: Attachment — Submitted filename: Nystrom_PLoSCompBio_response_to_reviews.docx [file pcbi.1008991.s001.docx]

**Comments to the Authors:**

**Reviewer #1**: This manuscript by Nystrom and McKay describes “memes”, an R/Bioconductor package that provides an interface to the MEME suite of tools for analysis of sequence motifs. While the package largely acts as a wrapper around existing MEME suite tools, the functions it provides will make it easy to incorporate these tools into an R-based reproducible analysis pipeline, including publication-quality visualisations thanks to the new visualisation functions included in the package. In addition, the fact that memes uses Bioconductor data structures to provide a familiar interface will make these tools accessible to a wide range of users by reducing the need for users to write custom code to format their data. Overall, the package should be a very useful tool to the many users of the MEME suite and Bioconductor.

I have only minor comments:

1. I would find it helpful to have a figure or table early in the paper with an overview of the tools available in the MEME suite, which of these are already implemented in memes, their purposes (i.e., de novo motif identification, motif enrichment, motif matching, etc), and perhaps a visual example of the output. There are many options available in the MEME suite/memes, so providing an overview early on would help give context and guide the reader through the rest of the manuscript.

Agreed, a table listing MEME Suite tools that are included in memes would provide a helpful overview early in the paper. We now include **Table 1** (below) in the revised manuscript.

We prefer to list only those tools that are included in memes in **Table 1** to maintain focus on memes’ capabilities. However, in our response to Reviewer #2, we have included a larger table that additionally contains a list of all existing MEME Suite tools, whether the tool is currently contained within memes, as well as the popularity of each tool (represented as the fraction of unique users that visit the MEME Suite website in order to use each tool). **84% of users** that use the MEME Suite webserver employ a tool provided by memes, indicating that the majority of user needs are met by the current version of memes.

***Table 1***. A list of MEME Suite tools that are currently included in *memes*.

| **Tool name** | **Purpose of tool** | **Description of output data** |
| --- | --- | --- |
| MEME | de novo discovery of ungapped motifs from user-provided sequences. | List of discovered motifs with estimated significance. |
| STREME | de novo discovery of ungapped motifs, optimized for large numbers of sequences. | List of discovered motifs with estimated significance. |
| AME | motif enrichment analysis from user-provided sequences. | List of enriched motifs with estimated significance. |
| Tomtom | matches user-provided motifs to databases of known motifs. | List of matching motifs with estimated significance for the quality of match. |
| FIMO | finds individual occurrences of target motifs in user-provided sequences. | Table of all motif occurrences with estimated significance. |
| DREME | de novo discovery of short ungapped motifs. | List of discovered motifs with estimated significance. |

2. Although most of the software and R/Bioconductor packages that are mentioned have references, there are no references included for R itself, the Bioconductor project, or the tidyverse packages. Given the tight integration with Bioconductor, the authors should consider adding a citation for at least the Bioconductor project.

The manuscript now includes references to R itself, Bioconductor, and the tidyverse packages. Apologies for the oversights.

R Core Team (2021). R: A language and environment for statistical computing. R Foundation for Statistical Computing, Vienna, Austria. URL [http://www.R-project.org/](https://www.r-project.org/).

Orchestrating high-throughput genomic analysis with Bioconductor. W.Huber, V.J. Carey, R. Gentleman, ..., M. Morgan Nature Methods, 2015:12,115.

Wickham H, Averick M, Bryan J, Chang W, McGowan LD, François R, Grolemund G, Hayes A, Henry L, Hester J, Kuhn M, Pedersen TL, Miller E, Bache SM, Müller K, Ooms J, Robinson D, Seidel DP, Spinu V, Takahashi K, Vaughan D, Wilke C, Woo K, Yutani H (2019). “Welcome to the tidyverse.” *Journal of Open Source Software*, **4**(43), 1686. doi: [10.21105/joss.01686](https://doi.org/10.21105/joss.01686).

**Reviewer #2**: In this paper, Nystrom et al. presented a R/Bioconductor wrapper (memes) for MEME Suite. MEME Suite is a well-known and well-maintained collection of tools (command-line and web-server) to discover and analyze motifs using DNA, RNA, and protein sequences. There are 17 different tools available in MEME Suite, from motif discovery and motif enrichment to motif comparison.

Although the authors did a great job by providing memes for the MEME toolset; however, it provides the interfacing to only 35% (6 out of 17) of the tools available in the MEME suite. Hence the title and abstract are misleading. Additionally, the authors provide some useful visualization functions, for example, to compare motifs analysis across conditions.

While it is true that only 6/17 MEME Suite tools are contained within memes, most user needs are met by these 6 tools. Below, we include a table listing all the tools currently included in the MEME Suite, the purpose of the tool, whether the tool is included in memes, the tool’s popularity (represented as the fraction of unique users visiting the MEME Suite website in order to use each tool), and a description of output data (for tools included in memes). As the table indicates, **84% of users** visiting the MEME Suite webserver employ one of the 6 tools included in memes (https://meme-suite.org/meme/meme-software/usage-reports/MAIN-YEARLY-usage-report.pdf). Thus, memes will be of considerable benefit to the motif analysis community even though it provides a wrapper to only 6 tools.

The Reviewer makes a good point that the title and abstract suggest that memes provides an interface to the entire set of MEME Suite tools. Therefore, we have changed the manuscript title to “Memes: a motif analysis environment in R using tools from the MEME Suite” to convey that memes includes only a subset of MEME Suite tools rather than a comprehensive interface. We have made similar revisions to the abstract and introduction, and we now include a table listing which MEME Suite tools are included in memes (**Table 1**, see response to Reviewer 1).

| **Tool Name** | **Description** | **Contained in MEMES?** | **Users (2020)** | **Rank** | **Description of output data** |
| --- | --- | --- | --- | --- | --- |
| MEME | de novo discovery of ungapped motifs from user-provided sequences. | yes | 43,213 | 1 | List of discovered motifs with estimated significance. |
| Tomtom | matches user-provided motifs to databases of known motifs. | yes | 28,382 | 2 | List of matching motifs with estimated significance for the quality of match. |
| FIMO | finds individual occurrences of target motifs in user-provided sequences. | yes | 9,114 | 3 | Table of all motif occurrences with estimated significance. |
| MEME-ChIP | motif analysis by combining the MEME, STREME, CentriMo, Tomtom, and SpaMo tools. | no | 4,976 | 4 |  |
| CentriMo | positional enrichment analysis of target motifs from user-provided sequences. | no | 3,031 | 5 |  |
| DREME | de novo discovery of short ungapped motifs. | yes | 2,822 | 6 | List of discovered motifs with estimated significance. |
| STREME | de novo discovery of ungapped motifs, optimized for large numbers of sequences. | yes | *new, replaces DREME* |  | List of discovered motifs with estimated significance. |
| MAST | identifies sequences with the best combined match to user-provided motifs. | no | 2,600 | 7 |  |
| AME | motif enrichment analysis from user-provided sequences. | yes | 2,250 | 8 | List of enriched motifs with estimated significance. |
| GOMo | associates GO terms with user-provided motifs in gene promoters. | no | 1,929 | 9 |  |
| GLAM2 | de novo discovery of gapped motifs from user-provided sequences. | no | 1,511 | 10 |  |
| MCAST | identifies clusters of user-provided motifs. | no | 469 | 11 |  |
| MoMo | motif discovery associated with protein PTMs from user-provided mass spec data. | no | 462 | 12 |  |
| T-Gene | target gene prediction from user-provided motifs. | no | 338 | 13 |  |
| SpaMo | identifies preferred spacing between target motifs in user-provided sequences. | no | 332 | 14 |  |
| GLAM2Scan | scans user-provided sequences for gapped motifs. | no | 215 | 15 |  |
| cispammer | no longer part of MEME Suite | no | 5 | 16 |  |
| GT-Scan | identifies optimal target sequences for genome editing experiments. | no | *new since 2020* |  |  |

While some may argue that we do not need a wrapper in the first place for a tool that is available as a command-line and as web-interface for users with less computational skills. Nevertheless, it is good to provide efficient wrappers in programming languages with a large user base (such as Python and R) for well-used and maintained tools, given the reason is well justified. However, on the flip side often the citation credit goes to the wrapper tool, not the actual tool that runs in the background and does the heavy lifting. But the authors in their code documentation are listing and asking memes users to cite relevant MEME tools, which is highly appreciated.

We agree that the tools doing the actual heavy lifting should get credit for data output. As the Reviewer points out, we cite relevant MEME tools throughout the manuscript. We also prominently cite MEME tools in the memes manual on Github (https://snystrom.github.io/memes-manual/).

The manuscript is adequately written with use cases, but the wording in the title and main text needs attention and re-phrasing. Throughout the manuscript, the authors used the word “complex” to describe MEME output, structure, and analysis. I have been a MEME user for several years and did not find it complex - but user friendly and easy to run and interpret the results. The six wrappers are nicely documented and easy to use in R. The source code is available on GitHub, Bioconductor, and also through a docker container.

Given running the MEME tools in the background and importing the output to R is trivial, which authors claim “complex” throughout the manuscript (as many of these tools provide text/xml outputs). Further, memes only provide an R interface to 35% of the MEME toolset, which does not make memes a R/Bioconductor wrapper for the whole MEME Suite. I think the current version of the manuscript and the software are not in shape for a standalone publication.

We agree that the MEME Suite of tools is user-friendly, easy to run, and provides results that are easy to interpret. These features have led to its incredible popularity within the motif analysis community. However, our point is that the data structure of MEME Suite tool outputs can be multi-dimensional and possess one-to-many relationships. This complexity reduces the ease with which output data are visualized or used as input data for other tools. Memes solves these problems by using structured data frames, allowing easy integration of output data with other motif analysis tools, base R workflows, and existing R-based data visualization packages.

In response to the Reviewer’s comments, we have revised the manuscript to reduce usage of the word “complex”, and instead describe the features of MEME Suite output data that limit how they can be used in motif analysis workflows in R.

**Reviewer #3**: The authors propose an R library for accessing MEME suite functionality. They provide a user with the instruments to convert their data in the required format and to work with MEME suite output. The package seems to be well-designed and is adapted to work with other common Bioconductor packages. It passes all CRAN and Bioconductor strict package requirements. In general, I see no reason why this package should not be published.

We thank the Reviewer for their time spent evaluating our work.
